# Supplementary material for: Patterns of Intron Gain and Loss in Fungi
Source: PLoS Biol. 2004 Nov 30;2(12):e422. doi: 10.1371/journal.pbio.0020422 (PMC532390; doi:10.1371/journal.pbio.0020422)
Supplement: Table S1 — Also available at http://genes.mit.edu/NielsenEtAl/. (4.3 MB ZIP). [file pbio.0020422.st001.zip › NielsenEtAl/html/828.html]

AN1494.1.NCU02052.1.MG03637.1.FG09406.1


```
 CLUSTAL W (1.82) Multiple Sequence Alignments - Introns Inserted


Sequence 1: MG03637.1	1579 aa
Sequence 2: FG09406.1	1505 aa
Sequence 3: NCU02052.1	1870 aa
Sequence 4: AN1494.1	1282 aa
Alignment Length: 1939 aa
Number Identitical Residues: 277 aa
Alignment Score (without introns) 17886


MG03637.1 	-------------------MEDASNSGGEPDLYAWVVMSQEVEVEVNFVERQLTGFATLS
NCU02052.1	--------MSGLENGDVALANMTNAPDPVAAAEPYAILRQEVELDINFREKSVSGICTFK
FG09406.1 	MAALAMPQAPEPLNHSLLDADDGTASVDNAPTLEYFVLKQEVNLEVNFRDKRIDGTTDIF
AN1494.1  	-----------------MPGVVDTPAGPSWPGLGFTVAHQKVELELDFASRSLKGKTEII
          	                       . .        : :  *:*:::::* .: : *   : 

MG03637.1 	IVPLNGDNHKFETVRIDARQMDIDTDKITVNGN-------------KVKAVYHDPYDELD
NCU02052.1	IYPLVP---DLEELVLDTRQCEIDVSNITVDGY-------------KTRAQAHDPYDRME
FG09406.1 	LVTFN---DKVDDIVLDAADCEIDTENVYVAELREINGDIVEGQKRKTVATYNDPYAKLS
AN1494.1  	IHPHYK---DLRYIRLNFRQGELKR--VTVSGK-------------VPLVKYTDPYESLQ
          	: .      ..  : ::  : ::.   : *                   .   ***  :.

MG03637.1 	IPDGFNWDGQQFHYQQKRIGQILTRTTPEVPVSVQSKSG--HSPSYGSLEVSLR-PEEIM
NCU02052.1	PPTNAQIGATQHHIMKKWMAPLLPETRRQAPIVERAEQLNISVPADGSLRIRLRPDALIQ
FG09406.1 	HPASWSLRADHHDIRRKRAQSLFSSRKKDVPAEHREFAG--CTPVYRSLKVNLRGKAEPD
AN1494.1  	LYGPHYHQRLASKLDALLKTPSEPELTLTLPKSVRIDELD-PFSVEAQDQMALRAPGLAD
          	            .          .      *   :    .   .   . .: **      

MG03637.1 	--VKKAGFKIRLKNNDFESSK-----VLQYDIVIPFRSRPFRDGFQFVGVEETDTRYPHF
NCU02052.1	DSEPKRILKIKTPKSVSENATGLSDPNEELTVVVPFRSKRIRDGLHFVGVEEGDLRYPHV
FG09406.1 	--RPRLIIRKSMLSTDSAEKT-----NKQYKITIPFTNVNPRDGIQFVGVDPLDNRFTHM
AN1494.1  	--DTEGPLSSKTAETSLPRFT-------ALTVTIEFIVENIRDGIQFVGVENGDKRYPHA
          	    .  :     ..     .          :.: *     ***::****:  * *:.* 

MG03637.1 	YTRNSEGLGMASCIFPCIDDPGARSYWTLKVKIPRTLGDALKQPLLTQR---ATTKNG--
NCU02052.1	YTRHSLEPGTASCIFPCLDDPGSRHSWTIKITCPRTLEDAFAPPLATQSALLTALDGGRK
FG09406.1 	YTRSSIHPGTSSCIFPCVDDHGSRCDWRISIKYPRTLGDALQQALATQQNGNNPDKMQ--
AN1494.1  	YTTNSLDHGTGCPLFPCVDDPSSRCTWEIAIRCPCTLGDVFERKPRDQSVSGTGTRLR--
          	**  *   * .. :***:** .:*  * : :  * ** *.:      *            

MG03637.1 	-----TDADESRFALTEEDKLLDMTVIASGILVGEHSDKDDDSKKTMEFQITGKEMSSQH
NCU02052.1	RKASDISAHQPGSSFTEEDKLREITVVCSGNLIGEQMHPKDERKKTMIFECV--NTAARH
FG09406.1 	-----IDGQERASYLAEEDKLREMSVVCSGFLMEEIVDPEDDHKKIMTFEPE-KKVSVQK
AN1494.1  	---------SNHDRLAPDDEALDLSVVCSGELTDDIVDSKDSSKKTVSFASY-SPLSARQ
          	         .    :: :*:  :::*:.** *  :  . .*. ** : *    .  : ::

MG03637.1 	VGFAVGPFQHVDLWSEFRTEADDDKLGKLAIKVHGYCLPRREDEVRFTCEPMSMAADFFA
NCU02052.1	IGFAVGPFEHVDLGAEFRTEEADEKLGANAARIHGYCLPGRVDEVRWTCQAVVAAADYFA
FG09406.1 	LGFAVGPFEHIDLSSEFRTEEDEMKLGMNALKVHAYCLPHRADWVRNTAAATTMAADFFT
AN1494.1  	IGFAVGPFEYVNL-ADFRESDQDEQLGQNAIPLHAFCLPGRGDEVRNTCFPMAKAIDFFS
          	:*******::::* ::** .  : :**  *  :*.:*** * * ** *. .   * *:*:

MG03637.1 	TKFGQYPFDSYKMCFVDDMITDTMPLCSFSLCSSRLLYPHDIIDNESEVTRELVHALASQ
NCU02052.1	PEFGRYPYEAYKMCFVEDLVHDTAVANSLSLCSTRLLYPETIIDTDIEVTRKLVHALASQ
FG09406.1 	FTFARYPFGNFKLCFLDDMVQDNVSFYSMAFISNRLLFPDDIIDTEIDVTRKIVQTLAYQ
AN1494.1  	MTYGSYPFSSYKMCFVDDAPEDTLPTACFSICSSRLLFPEEIIDPMYDSTRAITHALAAQ
          	  :. **:  :*:**::*   *.    .::: *.***:*. ***   : ** :.::** *

MG03637.1 	YFGINIIPDTISDLWVTIGISHYITDLFMQQLCGNNHYRFQMKTRADKLVEEDVNRLPLD
NCU02052.1	YVGVHIVPNERSDTWLIIGLQWFMTDLFMRTICGNNWYRFHIKSLSDKLVEADVRRPSLH
FG09406.1 	WIGINMIPNTRNDMWLIIGIAHFMTDLFMKKLCGNNEYRFRMKTLSDKLVQVDVDRPSLY
AN1494.1  	WIGVNIVPKEPADTWVTVGVAWYITDTFMRKLCGNNEYRFRLKLMSDRVCDLDYERPSVY
          	:.*::::*.   * *: :*:  ::** **: :**** ***::*  :*:: : *  * .: 

MG03637.1 	QLGYYLGLGDYQFEFMALKAPLVLFVLDKRMTKKASHNGVRRVVLRLVSRSNTEASD--V
NCU02052.1	ALGDYLHLGDFELEFMALKAPLVLYILDQRMSKIPGSTGVVRVLSQMVSAANISNTDPKA
FG09406.1 	DLGAYLHLGEFEMDFMALKAPVIFFILDKRLIKASGGHGLTRILSKMLTKVQIEGSDK-A
AN1494.1  	DMGNILSIDPSEAKFIALKAPLVLFILDRRLTKASGKATMSRIISRLFLNARMGELPN--
          	 :*  * :.  : .*:*****:::::**:*: * ..   : *:: ::.   .        

MG03637.1 	RFISSESFRKQCEKVHKGAPLELFWQQWVQSAGCARFDMSQRYNKKHSRVDLTVRQPEEK
NCU02052.1	TTLSFQDFRRWCEKRSQYV-PDELWNQYVFGAGCPRLDIHQKFNKKNLNVDIQILQYQG-
FG09406.1 	TILETEKFRATCEKGAKYR-LESFWNQWVYGSGCPRFDVKAKFNKKRLCVELTLNQIQS-
AN1494.1  	GAVTSSVFQKTCERLGHAK-LDTFFNQWIYGAGCPRFQATQRFNKKKLVVEMMIKQVQS-
          	  :  . *:  **:  :    : :::*:: .:**.*::   ::***.  *:: : * :  

MG03637.1 	SQLPQPPFNKNNFWGVLQDGRHVPAAKKENVSVWFTGPITIGIHEANGTPYEHVAEIQRT
NCU02052.1	--PTKKSISKDEFWRELQEEIH--QVYAGDVPKLFTGPFTVRIHEADGTPYEHYQLITEK
FG09406.1 	MMVKKAPLEKEDFLRVVKERR--SGVKAGEVQPLFTGPMTVRIHEADGTPYEHILEIRED
AN1494.1  	DQPTARDLERDAFMRDVKEEIR--NVYAGTIQPVFTGSMTIRIHEADGTPYEHIVEIKEG
          	       :.:: *   :::      .    :   ***.:*: ****:******   * . 

MG03637.1 	GPRQFKAIEMPYNTKYKRLKRTRRQKERAQALVQAGEGGEQAEEAVLYSLGDVLQSPEEE
NCU02052.1	DKG-GTNLQIAYNTKYKRLKRTKKAMAAAANSNSDKHEIQEDDVVYFNMLGDVLTSQKDM
FG09406.1 	ATR-STKFEIPYNTKYKRLKRTRRMKEKQN--VGASMDAENLDDTLLYCLGDVLQTPEEQ
AN1494.1  	VTK----FDIPYNTKYKRLKRNKRQKERAAAVIGADGNAEVQEDILLYCLGDVLQSEEEM
          	       :::.**********.::               :  :   :  ***** : :: 

MG03637.1 	KEWGLADWTPEQVKAMEQDSFEWLRIDADFEWICSMKTNMQPWMYVSQLQQDRDVVAQQE
NCU02052.1	EDWGLQDWASEVQTKMDQESYEWIRLDANMEWLCFMKTDMQEYMYLAQLQQDRDVVAHQD
FG09406.1 	AQWELIDWDPETERKMDQESYEWIRVDADFEWSCDMKRTLEPYMYVSQLQQDRDVVAQQD
AN1494.1  	QQWRLADWSKEDEERMGQESYEWIRMDADFEWICKLSLVMPGYMYLSQLQQDRDVVAQLE
          	 :* * **  *    * *:*:**:*:**::** * :.  :  :**::**********: :

MG03637.1 	~SVLWLSQKAGGEILATVLGRTLMDTRYYHGIRTQAIKALQKQVWTKEEESLDPDSGIVN
NCU02052.1	~AMLAFKREKRHAVHSTIETRTVMDRRYYHGVRVMALEDLAKQAHP-DLNYIG-------
FG09406.1 	~AMLYLTQGPLHPIASGFLTRTLVDRRYFHGIRTMAAEALPRQANIKDLSMLG-------
AN1494.1  	0SLQYMAAQREHPLISTIFLRTLMDRRYFYGIRVAAARALVKHAKE-EINWLG-------
          	 ::  :       : : .  **::* **::*:*. * . * ::.   : . :.       

MG03637.1 	KLKKTDAGGLRILMKAFKAFFCYPDSQTPR---------PNDFSNKMQYAVQRAIPEAIA
NCU02052.1	---------MVHLILCFRKFFCKKVPIPGREGGYHYPIAPNDFTDKGQYAIQCAIPAVLA
FG09406.1 	---------LRQLMKAFSEMFCHKGTNQPK---------PNDFSDKRQYNVQCAIIKAIA
AN1494.1  	---------LFHLEKAFQELFCLPGSPMTR---------SNNFSDRAAYVLQLVIPEAIS
          	         :  *  .*  :**   .   :         .*:*:::  * :* .*  .::

MG03637.1 	RVREKG---RCPREAQQFLLEQLQFNNNTGNE~YSDHFYVAGLLDALATSLIPDNSV-ET
NCU02052.1	RTTDLQEKGRCSKRARQFIMEMLLFNDNSENE~FSDQFYIAKLLDALTTSVIPQKEITEG
FG09406.1 	QVRDANTY-KCPLEARQFILDQLLFNNNEDNP~FSDHFYIATLVEALATSLIPSKQD-DW
AN1494.1  	KVRDVSG--KTPMRVKRFLYDKLKFNDNSNNE0YSDNFYVATLMKSLCHAMLGRVES-RT
          	:. :     : . ..::*: : * **:*  *  :**:**:* *:.:*  :::   .    

MG03637.1 	VTLRNSD--DPETALDEEFLEA-ALQMIERYLRMDEWTNSYQNIWTVTALGCKQKLMKAG
NCU02052.1	YLIKNLDPFDEDDAEFKSFIEK-TIEEIDKFRRMDEWTLTYQNIWTTTALHCKMRLMKAR
FG09406.1 	FAMQN----KIPNEEEKQFLEK-AIEQIERVLRRDEWTHSYQNVWTIAGLSAKQRLMKAD
AN1494.1  	DDLNNFDMDAVLEAQAEEQLEKDAIAEIDRYRRMDEWSSSFQNLYSRAALYCQMQLMQAK
          	  :.* .         :. :* .::  *::  * ***: ::**::: :.* .: :**:* 

MG03637.1 	VIPISAIDFVRYLQDSTLDLISIKAFEALVDLGFLFNKKILRLLLAYMTTHYSPFMRDRL
NCU02052.1	VIPESALDFVQYLQDDNIDLVRIKAFECLVELGMISKPPILKLLLSYMSTDSSPFIRDRL
FG09406.1 	VIPKKYGDFGQYLLDGTRDLIRIKAFEGLIDLGAMLDPTVFSFLTYALITDRSPYVRNKL
AN1494.1  	IAEVDVMQFLPYTRVGTYDLLRLDAFECLVELDIFKSPELLRWFIFTMSNDPSIWLRWQL
          	:   .  :*  *   .. **: :.*** *::*. : .  ::  :   : .. * ::* :*

MG03637.1 	FKVFAWGVAGLAFGEYDELKSKAEQQAKLKEDDEAEDDLEGDSLMLVNADAILERKKAKE
NCU02052.1	FKVFCRGIAAIAFGEN-------------KASTQELEALNDGGLVIDQGDAEIQQRKLDA
FG09406.1 	IEAAASGLAAIAFGEH----------TKVTKNESPPEDEEGDLLLVQDSAQEIEARKEMF
AN1494.1  	HGLFGRALAPVAFGRG--------------VINEPPPPTDG---LIIEQESSTEVRQADL
          	      .:* :***.                  .     :.   :: :     : ::   

MG03637.1 	ARRTTVGAAVAELRKEFGKTERQD---LMIEIWRAATSPIIGLKEQRSLLDMCETLFEAD
NCU02052.1	VRREDIAHALNALKEELRGN-----TEMQISIWKAFNSPVIGLQEKRQLLELCSAMFEPE
FG09406.1 	ARKENLDAALKALRREMEQTYGGDERHYSTAMRKALDHPDLGRGEMESMLDLAAMMFEEA
AN1494.1  	ARRQTVTGALAALKAEVSGD-----QVLKECLWAACSSPIIGILELSEFTDLCRVIYDPV
          	.*:  :  *:  *: *.              :  *   * :*  *  .: ::.  :::  

MG03637.1 	YSCRVTVRHPTKWHCERGPTEPKKLVIHF-----KA~VQRKITKRPEPKPE-------ET
NCU02052.1	DSLLVTIKYPIVWKATRDPTIVATAPQILGTPPKKR~CVVKFKSEYRTLPKSKDMVAIEA
FG09406.1 	GDWVVTVPLPKAWSVER----ERPVQQLS-----NR0LIMKFKAHYKTKPK-------DP
AN1494.1  	TSKRVTLKYPRYWAVKHHG----------------R0GRLQFYRTNKIRTS---------
          	 .  **:  *  *   :                       ::    .  ..         

MG03637.1 	PVKPVAVPAVVPT----------------------------------VTPAAAPPTSAPT
NCU02052.1	PPPPAPLPAPTPSTNLSASRVEIKPPEKKTIVKLQSRPSYIAKPRESPVPEAPPPVRKDS
FG09406.1 	VVQPLPLPAAAP-----------------------------------VAPIAAP------
AN1494.1  	------------------------------------------------------------
          	                                                            

MG03637.1 	PTAALSHTPMEGV-------VATPTMPAPPQRQGSTKIKFNVNKGVDKSRQNSTTPLPKA
NCU02052.1	IAMTVPPRPSTTAGPRTPLPTSVPSAPAPNTSAAQPSIKTAVPKAASPPPVPAPKPQQSN
FG09406.1 	---------------------AEPKRPVPLQKTSS--IKINTHK---------TAPPQRP
AN1494.1  	---------------------LNPSTTSSAKRKRE------------------------D
          	                       *. . .     .                         

MG03637.1 	STTLPKPSTA---LPKVSTTLPKPSTTLPKVSTLPKVSTLPKVSSTVSKVSSTASAIAES
NCU02052.1	DVAVPRPSTVNLGKPKIVKPVIKRKSDESFENPSPKKIIKTNSDSFRSDPSRNSSFKTEP
FG09406.1 	SVPPQRPSIA---APVQS---PKPSQTKGERDTIVASPVLSRQPSTSSSSGSKPSWPPEP
AN1494.1  	GTAMPAPSSG------------------------LRVTFKQSRPNPSNHHPSTPSAKTPG
          	...   **                                    .  .    ..*  .  

MG03637.1 	PSTKVLGGSISSSPSTMQPTSTPTTQRTALPSGS-ISGNGALAKKPSKLISTPVNGSIKR
NCU02052.1	PRSESFKKEAPRSEAIRGHSFRESAPQIPPPRTSSFSVRPNANGQPSKVVTLPFRKWTKL
FG09406.1 	P----------SSTNVKRPRPDKDEHHTPAP------------KRPK----------VEK
AN1494.1  	P-----------------------------------------------------------
          	*                                                           

MG03637.1 	KLDDASGPPSKSPRPTKIVRVRLPQYINKLRPDTRQQILR-ENALKATSSRQ----HSST
NCU02052.1	SARAQRSIDYEQSQREKQMLAASARGISGSLATPVHKRSSAAVGFGSGSAPSSPKSRMQS
FG09406.1 	PFGADSGVAAKKKKRRMVRLKVTPHRLASVMKDQKKPIISGRTSLPSGGSRDGPSLLSNS
AN1494.1  	----SSGLGTPLQQRQPTKLHIPNFAAAQVHRQPP-------------------------
          	      .      :                                              

MG03637.1 	GD-------GSITAKPAR------------------------------------------
NCU02052.1	PAGNGYGSSGSYAEKEQRNKERDRDRERDRDRERDSSSKFREKDRNDHDRERYRGGAERE
FG09406.1 	RS-------DSITAKPAR------------------------------------------
AN1494.1  	------------------------------------------------------------
          	                                                            

MG03637.1 	---------------------------------------------------KPLPSGSAP
NCU02052.1	KEKYRERERDRESDREHERPREKERDREKYRERGDRDSDRERDRPREKYREKGTDRGDSD
FG09406.1 	---------------------------------------------------KPLPTGDAA
AN1494.1  	------------------------------------------------------------
          	                                                            

MG03637.1 	TSSSTPQQQKVRKPLPN-------------------------------------------
NCU02052.1	RGGRTTDSDRERNRIDRDRDRDRERERERSDRDRERTDRERERIRDKYRDRADRDSDRDR
FG09406.1 	RRPLP--GGPASSPPER-------------------------------------------
AN1494.1  	------------------------------------------------------------
          	                                                            

MG03637.1 	---------------------------------------------KSSPATTSVSEKKEA
NCU02052.1	DSYKDRDRERDRDRDRDRERDRGDRGDRGDRDRDRDRDRDRGDRDRVSSSGSSLLIKKDR
FG09406.1 	---------------------------------------------KISLSTHSNSTPK--
AN1494.1  	------------------------------------------------------------
          	                                                            

MG03637.1 	PPALPPLDPVRAVQAQ-------------SATAASPSPSSGTK--------LKIKFKVK-
NCU02052.1	KPLPGSGEKERRPLPTGAPHEKAPHPLKESHSPIHPTPAATTNGGEPKKKIIKLKLKSSG
FG09406.1 	--------PKSASPAT-------------STPPPGAPPAPRTK--------IKIIRKSN-
AN1494.1  	-----------------------------SHTPATPSTPGGG---------LKLKLKLG-
          	                             * ..  ....            :*:  *   

MG03637.1 	--------PSQPPPQ-------
NCU02052.1	SGLGGGSGSSSTPGAGGGSGSR
FG09406.1 	--------PQPPPAS-------
AN1494.1  	----------SQPKQ-------
          	            *
```
